# *Saccharomyces cerevisiae* biofactory to produce naringenin using a systems biology approach and a bicistronic vector expression strategy in flavonoid production

**DOI:** 10.1128/spectrum.03374-23

**Published:** 2023-12-13

**Authors:** Luis Alberto Mejía-Manzano, César Iván Ortiz-Alcaráz, Laura E. Parra Daza, Lina Suarez Medina, Teresa Vargas-Cortez, Miguel Fernández-Niño, Andrés Fernando González Barrios, José González-Valdez

**Affiliations:** 1 School of Engineering and Science, Tecnologico de Monterrey, Monterrey, Nuevo León, Mexico; 2 Department of Chemical and Food Engineering, Grupo de Diseño de Productos y Procesos (GDPP), Universidad de los Andes, Bogotá, Colombia; 3 Department of Bioorganic Chemistry, Leibniz-Institute of Plant Biochemistry, Halle, Germany; Lerner Research Institute, Cleveland Clinic, Cleveland, Ohio, USA

**Keywords:** naringenin, *S. cerevisiae*, 2A peptide, systems biology, bicistronic vector

## Abstract

Naringenin is the central flavonoid in the biosynthesis of several bioactive compounds and presents a growing demand for its nutraceutical properties. Naringenin extraction from plants is non-viable due to low yields, and microbial platforms could represent a controlled and sustained alternative to produce it using several metabolic engineering tools. This study shows the naringenin production in *Saccharomyces cerevisiae* from glucose through a combined approach of systems biology, enzyme criteria selection, and a molecular engineering strategy. *In silico* prediction using a mixed integer linear programming (MILP) algorithm showed that the phenylpropanoid pathway was the shortest and most viable metabolic pathway. Two biscistronic constructs were generated using the PTV-1 2A peptide sequence, and a naringenin biofactory was assembled with the phenylalanine ammonia-lyase/tyrosine ammonia-lyase genes encoding phenylalanine/tyrosine ammonia-lyase (*Rhodobacter capsulatus*), 4-coumaroyl (4 Cl) encoding a *p*-coumaroyl-CoA ligase (*Solanum lycopersicum*), CHS encoding chalcone synthase (*Hypericum androsaemum*), and CHI encoding a chalcone isomerase (*Glycine max*). Naringenin productivity in batch fermentation was about 40.67 ± 3.47 µg/Lh with a 6.10 ± 0.52 mg/L titer (22.41 ± 1.91 µM) and a 3.26 ± 1.36 mg/g yield (*Y*_*P*/*S*_) with the detection of additional flavonoids. The obtained concentration is better than other related works in diverse engineered microorganisms. The results suggest a successful and optimizable alternative for the heterologous flavanone production in yeast combined with bicistronic expression mediated by a 2A peptide sequence for the first time. This strategy supports the production of extensive routes for other nutraceutical compounds.

**IMPORTANCE:**

Flavonoids are a group of compounds generally produced by plants with proven biological activity, which have recently beeen recommended for the treatment and prevention of diseases and ailments with diverse causes. In this study, naringenin was produced in adequate amounts in yeast after *in silico* design. The four genes of the involved enzymes from several organisms (bacteria and plants) were multi-expressed in two vectors carrying each two genes linked by a short viral peptide sequence. The batch kinetic behavior of the product, substrate, and biomass was described at lab scale. The engineered strain might be used in a more affordable and viable bioprocess for industrial naringenin procurement.

## INTRODUCTION

Flavonoids are phenolic compounds of two six-membered rings and a three-carbon unit with diverse attributed biological and pharmacological properties like antioxidant, antiinflamatory, antitumoral, antineoplastic, antithrombogenic, antiatherosclerotic, antidiabetic, cardioprotective, hepatoprotective, antiviral, antibacterial, and antifungal activities (1, 2). Naringenin (5,7,4'-trihydroxyflavanone) is a flavanone, considered a central molecule in the biosynthesis of other flavonoids, and like other molecules in this group, it presents interesting biological and therapeutic activities (3, 4). These types of compounds are exclusively biosynthesized by plants; however, their industrial exploitation presents problems such as long growth periods, low abundance, and complex purification steps (5). To date, chemical synthesis of naringenin and other flavonoids is unfeasible, and a viable alternative for overcoming the described difficulties and to obtain these compounds in economic and environmentally friendly ways is through their production in genetically modified microorganisms, using them as cellular biofactories (6). To achieve this, systems metabolic engineering plays an essential role in integrating several methodologies and tools derived from the metabolic engineering, systems biology, and synthetic biology fields (7).

Until now, heterologous production of naringenin has been carried out mainly in *Escherichia coli* and *Saccharomyces cerevisiae* strains and in a minor degree in *Streptomyces* sp. (8). In these microorganisms, the needed enzymes of the phenylpropanoid pathway presented in plants and some microorganisms have been expressed. Basically, the mentioned pathway starts with the tyrosine or phenylalanine amino acids, which are converted into p-coumaric acid. In the case of phenylalanine, this change is catalyzed by two enzymes: phenylalanine ammonia-lyase (PAL) and cinnamate 4-hydroxylase (C4H), while for tyrosine, the reaction is only carried out by tyrosine ammonia-lyase (TAL). After that, *p*-coumaroyl-CoA ligase [4-coumaroyl (4 Cl)] forms *p*-coumaroyl-CoA, and then three molecules of malonyl-CoA are condensed, leading to naringenin chalcone by chalcone synthase (CHS). Finally, the chalcone isomerase (CHI) renders naringenin (9). The use of diverse organisms as a source of enzymes and even isoforms reflects the diversity in the productivity results. Despite the advances in the use of additional genetic engineering strategies to achieve the best naringenin productivity, production yields have remained low (10), and the need to continue generating stable engineered strains with minimal alterations and maximal efficiency is demanded.

The use of yeasts over bacteria as hosts for flavonoid production is advantageous since yeasts present limited interference of the endogenous secondary metabolism, subcellular compartmentation, and the ability to perform post-translational modifications required for eukaryotic enzymes (11). Particularly, the genome of *S. cerevisiae* is widely sequenced and known, with a diversity of studied tools for its alteration and prediction. Moreover, it is recognized as a generally recognized as safe (GRAS) microorganism in the pharmaceutical, food, and nutraceutical industries (12, 13). Nevertheless, one of the scarcely mentioned causes of *S. cerevisiae*’s low performance when enzymes of complex pathways requires to be expressed is its low tolerance or instability for the transcription of multiple heterologous genes (11), in addition to the size limitations imposed by the vectors used (14).

In this regard, the discovery of 2A peptide sequences can help tackle these difficulties. 2A peptide-like sequences or *cis*-acting hydrolase elements (CHYSELs) are small aminoacidic (18–22) peptides found in some viruses, which can be harnessed as mechanisms for expressing several proteins in a single transcript. The ribosomal complex skips the 2A peptide, and the translated fused proteins suffer “self-cleaving” (15, 16). In literature, this described system has begun to be studied in multi-gene expression in several organisms (17 – 19). In this work, we present the development of an *S. cerevisiae* cell factory to produce naringenin, supporting the choice of the biosynthetic pathway by predictive algorithmic analysis and the testing of the multi-gene co-expression through 2A peptides.

## RESULTS AND DISCUSSION

### Construction of the *S. cerevisiae* naringenin-producing biofactory

**Fig 1:**
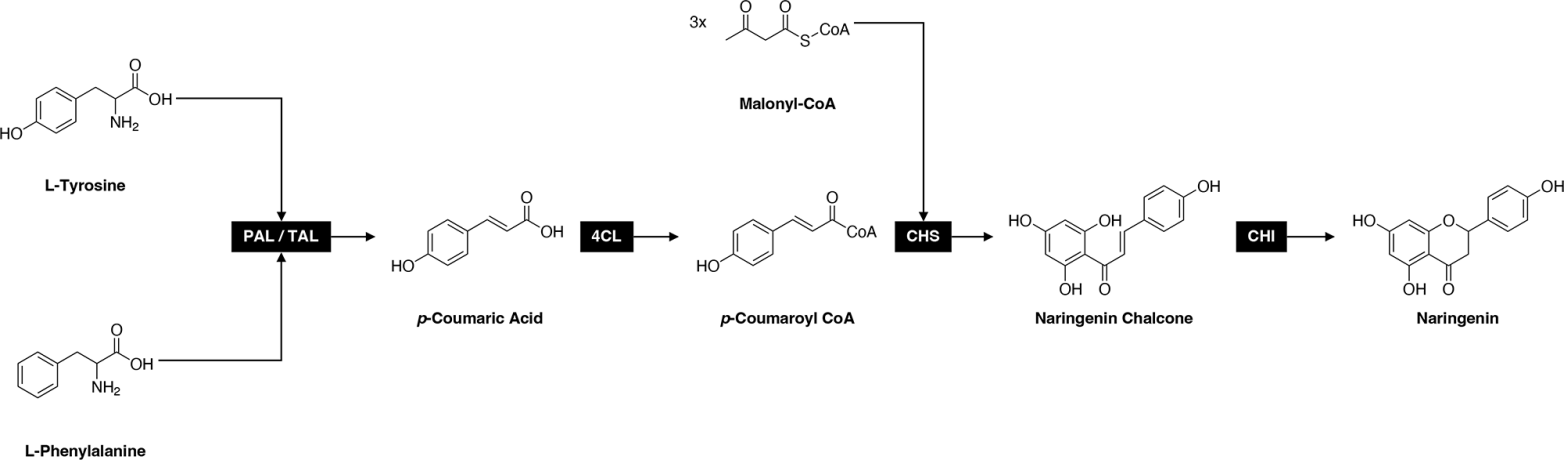
Expressed phenylpropanoid metabolic pathway for naringenin production.

We constructed a naringenin-producing biofactory with *S. cerevisiae* based on the determination of the shortest metabolic pathway for naringenin biosynthesis and gene and enzyme selection. The selected phenylpropanoid metabolic pathway is schematized in Fig. 1, and it involves the use of four key enzymes in the naringenin biosynthesis from L-tyrosine and L-phenylalanine (9). Gene selection obeyed the inclusion of three specific criteria [previous evidence of expression in yeast (*S. cerevisiae* preferably), a low value of the Michaelis-Menten constant (*K*_*M*_), and the existence of mutations for naringenin production]. The evidence of the previous gene expression in *S. cerevisiae* reported in the literature was considered the most important since this is a proof of successful expression of the enzymes. For the PAL/TAL enzyme, the three criteria were fulfilled by choosing the gene from *Rhodobacter capsulatus* which presents a high selectivity by L-tyrosine (*K*_*M*_ = 0.0156 mM) without losing its affinity by L-phenylalanine (*K*_*M*_ = 1.277 mM) (20) and with wide evidence of its expression in *S. cerevisiae* (13, 21). The 4-coumaroyl-CoA ligase (4 Cl) gene was taken from *Solanum lycopersicum* mutant Q274H since this enzyme, despite having a minor *K*_*M*_ (0.179 mM) value with respect to that of *Ruta graveolens* (*K*_*M*_ = 0.0031 and 0.0055 mM) (22), showed an increased naringenin production due to its mutation (23). For the CHS gene, the suggested options from the database (22) were *Hypericum androsaemum* and *Medicago sativa*, both with very similar *K_M_* values (0.0231 and 0.0227 mM, respectively). However, CHS from *H. androsaemum* was preferred because of the strong evidence of its expression in yeast (24). *Glycine max* and *Scutellaria baicalensis* were the candidates for the CHI enzyme gene according to the BRENDA database. However, attending to the absent reported Michaelis-Menten constant for the CHI from *S. baicalensis*, *G. max* was the chosen option (*K_M_* = 0.021 mM) (25).

The naringenin producer strain was constructed in two steps. First, the genes for PAL/TAL and 4 Cl enzymes linked by a PTV-1 2A peptide sequence were integrated to the genome of *S. cerevisiae* CEN.PK 2–1C, obtaining the IM90 strain. In a subsequent transformation, the CHS and CHI genes also linked by the 2A peptide sequence were incorporated to a IM90 strain to generate the IM100 strain. Both sets of genes were under the control of the constitutive TEF1 promotor and ADH1 terminator. Figure S1a and S1b show the growth of transformed colonies on selection medium lacking uracil or uracil and leucine, correspondingly. Also, these assays showed that there was no yeast growth in both negative transformation controls, in which the plasmid DNA and the plasmid DNA and single-stranded salmon sperm DNA (ssDNA) carrier were omitted, respectively, in the first and second controls. Colony PCR analysis confirmed the presence of the amplicons in each one of the colonies observed in the plates (data not shown). Later, for the first transformation, genomic DNA was extracted and analyzed by gel electrophoresis, indicating that the presence of the 2.2-kb amplicon for all the analyzed transformants (Fig S2a) in correspondence with the plasmid control is considered as a positive control of the amplicon (Fig. S2a, lane 3). The unmodified *S. cerevisiae* strain was also included in the assay, and it did not display the amplicon band. In the same way, for the second transformation, plasmid DNA extraction and electrophoresis tests revealed the expected amplicon of about 0.65 kb in the selected colonies, using pUDIB2P3 as a positive control (Fig. S2b, lane 6).

The efficiency in the first transformation had an average of 641 CFU/μg of plasmid DNA, and in the second one, the average was 5,600 CFU/μg. These low transformation efficiencies were lower than expected when using this modified lithium transformation method (26), but it could be caused by a genetic component in the strains and/or some other factors such as cell washing (27).

### Naringenin identification

**Fig 2:**
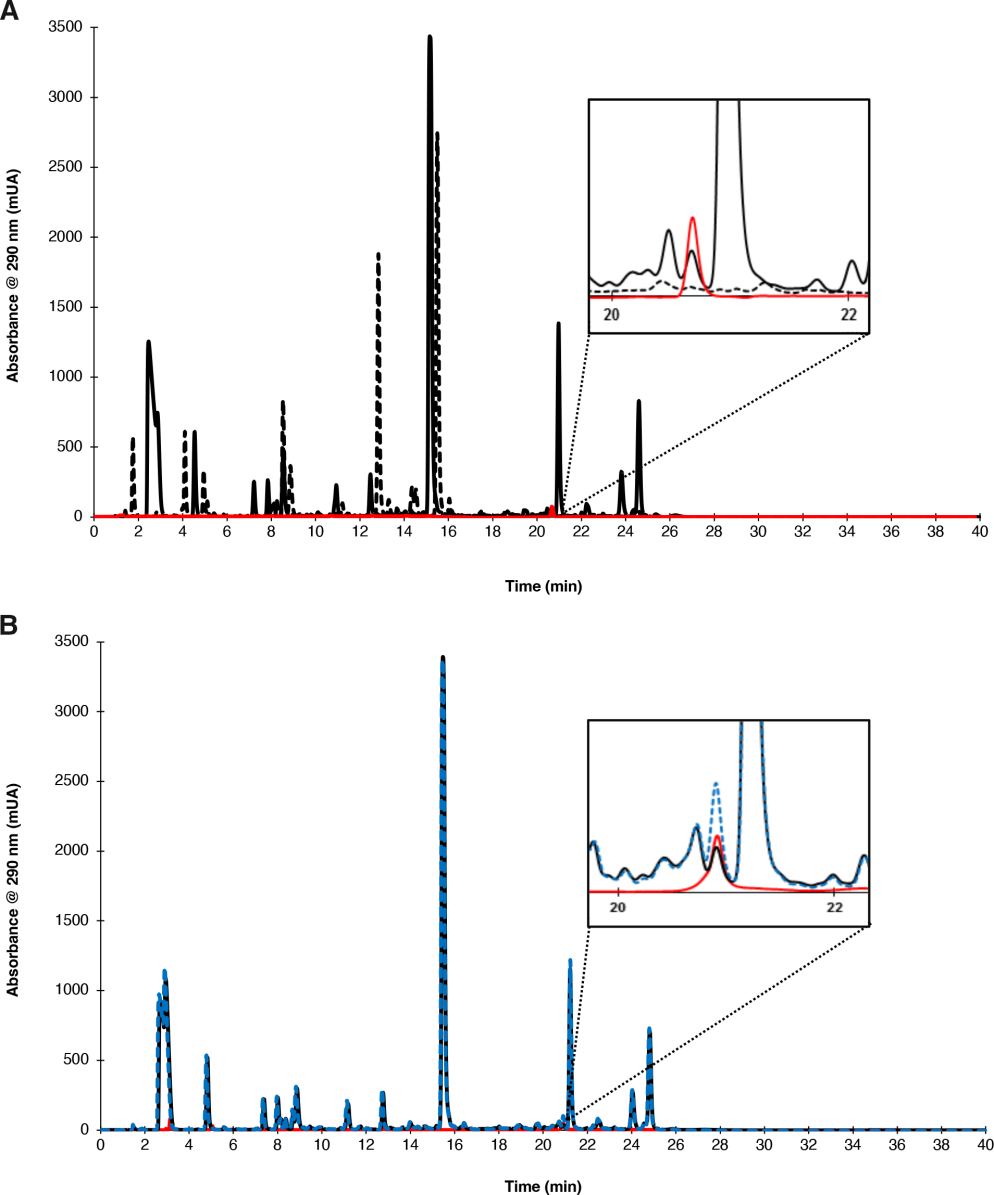
Identification of naringenin by HPLC-UV analysis. (**A**) Chromatographic profile of the engineered and the unmodified *S. cerevisiae* extracts. IM100 strain, continuous black line; unmodified strain, dashed black line; naringenin standard, continuous red line. (**B**) Chromatographic spiked profile for the engineered *S. cerevisiae* extract. IM100 strain, continuous blue line; spiked IM100 strain, dashed blue line; naringenin standard at 2.5 µM, continuous red line.

**Fig 3:**
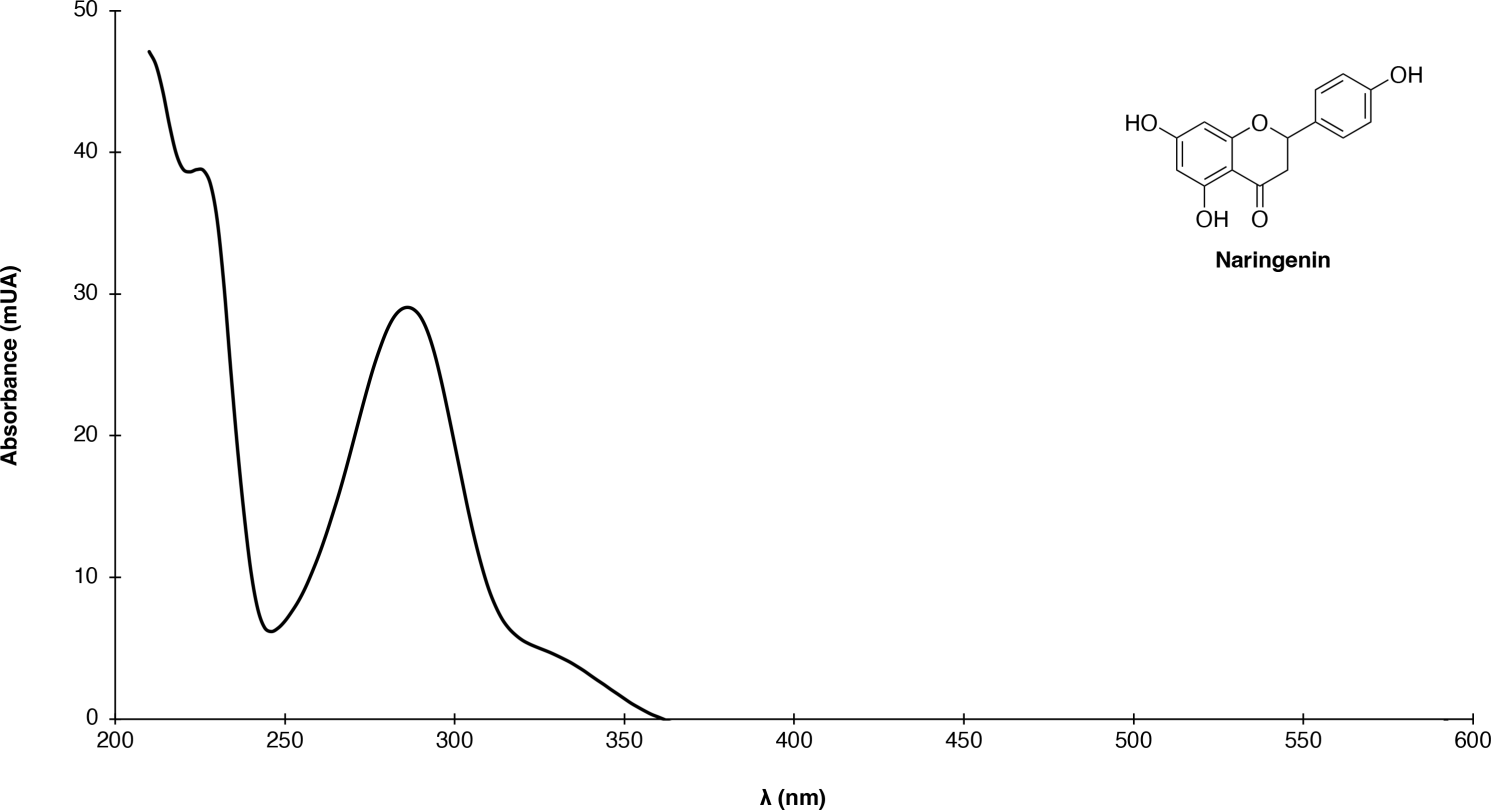
Absorption spectrum of identified naringenin at retention time (20.67 min) in culture with the engineered *S. cerevisiae* strain IM100.

Naringenin biosynthesis was verified by high-performance liquid chromatography (HPLC) analysis after culture media extraction. No naringenin formation was identified in the control extract experiment from the unmodified strain, while the compound was detected in the IM100 strain extract harboring the expressed genes (Fig. 2A). The identified peak had the same retention time (*t*_*r*_ = 20.67 min) in our analysis conditions as the naringenin standard. For additional verification, the sample extract was fortified with a naringenin standard at 2.5 µM. As a result, a proportional increment in peak area was observed (Fig. 2B). Also, the UV-Vis spectrum of the sample correlated adequately with a previously reported spectrum of naringenin in literature (28) (Fig. 3). Interestingly, about five additional hydrophobic compounds with absorbances at 290 and 230 nm were observed in the chromatographic profile of the engineered strain in comparison with the profiles of the unmodified one (Fig. 2A). These compounds with close retention times (21–26 min) to naringenin may be related to flavonoid derivates (such as pinocembrin, eriodictyol, homoeriodictyol, and phloretin) as a result of the biotransformation processes in the fermentation after flavanone biosynthesis (24, 29), since the majority of UV-Vis spectra of these chromatographic peaks (data not shown) presented the characteristic flavonoid bands in the range 240–285 nm (Band II) and 300–400 nm (Band I). Band I corresponds to the absorption of the cinnamoyl system in Ring B, while Band II is attributed to the benzoyl system in Ring A (30). However, further identification and characterization of these compounds are required.

### Bicistronic expression of the heterologous pathway of naringenin

Molecular evidence of the transformation and naringenin detection allowed the confirmation that the bicistronic vector strategy was suitable for expressing the phenylpropanoid pathway in *S. cerevisiae*. In this way, this work represents additional evidence about the suitability of 2A peptide sequences for supporting multi-cistronic gene expression. Some works have previously established the application of 2A peptide-like sequences in the efficient display of heterologous unrelated proteins or enzymes such as Sun et al. (31) and Subramanian et al. (18), in which a double lipase B from *Candida antarctica* was expressed in the membrane of *Pichia pastoris* and a cellobiohydrolase enzyme from *Penicillium funiculosum* and green fluorescent protein were assembled in the same cassette in *Trichoderma reesei*. In both cases, the 2A peptide of the foot-and-mouth disease virus (FMDV) was used. As it can be inferred, the testing of this tool for building compound biofactories remained unclear, mainly when a final product is the result of several expressed enzymes. For this reason, the present study contributes as positive evidence of this last aspect. Another of the studies holding the use of this system as an expression mechanism for complex metabolic pathways is the co-expression of the protein genes *ctrI*, *ctrE*, and *ctrYB* of the *Xanthophyllomyces dendrorhous* fungi in *S. cerevisiae* for β-carotene production (32). In this case, the genes were separated with a T2A sequence from the *Thosea asigna* virus. Also, friedelin production in *S. cerevisiae* was achieved with a bicistronic expression of friedelin synthase from *Maytenus ilicifolia* and 3-hydroxy-3-methylglutaryl coenzyme A reductase (HMG1), linked by the 2A peptide from equine rhinitis B virus (33). Also, the T2A sequence from *T. asigna* was recently used for expressing a four-copy cassette of the antimicrobial peptide plectasin with outstanding yields in yeast (34). For naringenin, this is the first time that production is achieved through bicistronic expression, which represents an important issue and advance in the heterologous production of this nutraceutical.

### Naringenin production and kinetics in batch cultures

**Fig 4:**
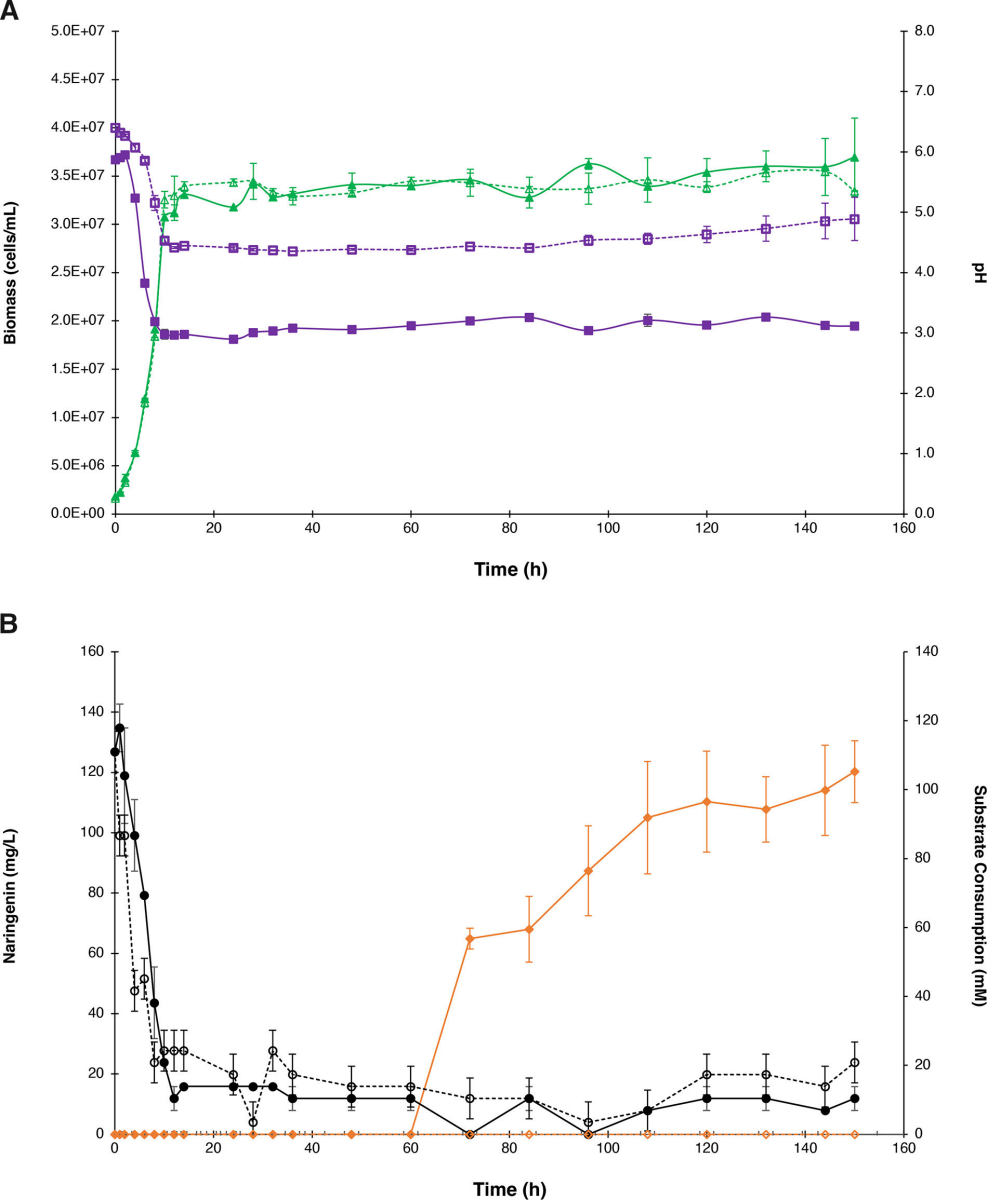
Growth and product formation of the IM100 and unmodified *S. cerevisiae* strains. (A) Growth curves of engineered and unmodified *S. cerevisiae* strains and culture media pH. (B) Naringenin production and substrate consumption of the IM100 and unmodified *S. cerevisiae*. Solid symbols represent the engineered IM100 strain, while open symbols indicate the unmodified strain. Biomass, triangle; pH, square; naringenin, diamond; glucose, circle.

Figure 4A depicts the growth curves for biomass and pH media of the batch cultures during 150 h of the engineered IM100 and unmodified *S. cerevisiae* strains. As it can be appreciated, under the cultural conditions, both strains presented similar behaviors in their growth. In fact, during the exponential phase, the IM100 presented a growth rate of 0.22 ± 0.02/h, very similar to that of the unmodified strain (0.21 ± 0.01/h) at a substrate concentration of about 4.5 g/L. Nevertheless, the medium’s pH in IM100 suffered a notorious decay from the beginning up to 10 h, and it was closely kept at pH 3.0 over the monitoring period. On its part, the control strain had a pH of about 4.4. This marked difference in the fermentation media acidity of the engineered strain compared to the unmodified strain can be explained by a more intense metabolic activity because of the need to produce naringenin flavone and consequently the generation of many acidic metabolites (i.e., trycarboxylic acid cycle [TCA] intermediate metabolites such as malate, citrate, fumarate, and succinate) in comparison with the wild-type strain (10).

When the substrate consumption is revised (Fig. 4B), a slightly faster consumption is observed in the unmodified *S. cerevisiae* strain than in the modified one at the beginning of the fermentation. However, in the next hours, the IM100 culture had a small concentration of glucose, being approximately half of the substrate concentration presented in the unmodified *S. cerevisiae* culture at the end of the fermentation. Glucose was consumed in 90.6% by the IM100 strain. During the first 60 h of culture, naringenin was not detected, although naringenin production has been reported to begin in shake flasks when the substrate is consumed in its majority (10). In this case, major glucose consumption reached a plateau around 36 h (IM100 strain). The late naringenin detection may be due to the low-level production (discussed later) and to the sensitivity of our developed analytical method. The concentration of naringenin at the end of the fermentation was 6.10 ± 0.52 mg/L (22.41 ± 1.91 µM), with productivity of 40.67 ± 3.47 µg/L/h and a yield (*Y*_*P*/*S*_) of 3.26 ± 1.36 mg/g. The obtained titer exceeded that in *E. coli* (35) and *Streptomyces venezuelae* (36) using sugars as carbon source. When the reports where *S. cerevisiae* is used as a host for this purpose are carefully reviewed, our result is more like the values achieved (i.e., 7 mg/L) when L-tyrosine or phenylalanine is fed (24), and it was higher than those cases in which the phenylpropanoid pathway has been only partially expressed starting from cinnamic acid (i.e., 200 mg/L) (19). The highest reported production of naringenin in *S. cerevisiae* has been up to 100 mg/L starting from glucose (13, 18) and up to 650 mg/L from p-coumaric acid (37); nonetheless, this titer has been achieved after strain and culture condition optimization. As it has been found from diverse reports (38, 39), low naringenin biosynthesis may be caused by the bottlenecks that the microorganism faces during flavonoid production. One of these is the limited precursor generation and availability in the cytosol, such as malonyl-CoA, which consequently interferes in the biosynthesis of the flavonoids (40). Moreover, the reported naringenin titer and production in our fermentation might be subestimated, and the value may be higher than the quantified, due to equivalent amounts observed in the other biotransformed flavonoid signals which are not being considered.

In our case, future production optimization is being considered. This might contemplate some metabolic engineering issues with intuitive approaches such as redirection of the carbon flux to target products (i.e., reducing intracellular concentrations of precursors by transporters, eliminating genes of precursors, overexpressing by-product genes, or increasing precursor supply by catabolism in other pathways), implementing flavonoid glycosylation and systematic approaches such as expression of multiple gene copy number or testing effective promotors (6, 41 – 43). Until now, through our current engineering design, naringenin production has been ensured by the sides of adequate custom-made DNA in the host, efficient affinity of enzymes for their substrates, and multi-cistronic expression. In addition, optimization at the bioprocessing fermentation level such as bioreactor culture and medium conditions (44) may give high enough titers even without appealing to metabolic tools, but this also requires further validation.

### Conclusions

This work presents, for the first time, the biosynthetic production of naringenin with the use of multi-cistronic expression through a 2A peptide strategy, starting from glucose without precursor use in an engineered *S. cerevisiae* strain. This represents additional proof of a successful case of the use of 2A peptide sequences for the heterologous expression of multiple genes. The expressed phenylpropanoid pathway resulted in integrative systems biology analysis through a mixed integer linear programming (MILP) algorithm and a rational enzyme criteria selection. The production of naringenin was verified by standard fortification in HPLC, and some other flavonoids were visualized at the end of the fermentation. There were no growth rate differences between the unmodified and the engineered *S. cerevisiae* strains in flask cultures. However, slightly lower changes were identified at substrate-level consumption and medium pH in the engineered IM100 strain compared to the unmodified one. Naringenin titer reached a value of 6.10 ± 0.52 mg/L, superior to some works producing this flavanone with *E. coli* and *S. venezuelae* used as substrate sugars and, at the same time, a comparable concentration in *S. cerevisiae* from L-phenylalanine or L-tyrosine. The previous analysis suggests strong and positive evidence of successful application of the bicistronic expression strategy using 2A peptide sequences. It is believed that this titer is decreased by the biotransformation effect of other flavonoid compounds observed at the end of the fermentation. However, genetic, metabolic, and bioprocess optimization will give important improvements in the production of this important flavonoid soon.

## MATERIALS AND METHODS

### Chemicals, strains, and maintenance

All restriction enzymes and DNA ligase were purchased from New England BioLabs (Massachusetts, USA). The solvents used in the chromatographic analysis were acquired from Desarrollo de Especialidades Quimicas (DEQ) (NL, México). *E. coli* XL1-Blue was provided by Agilent (Santa Clara, CA, USA) while *S. cerevisiae* CEN.PK 2–1C 30,000A (MATalpha ura3 his3 leu2 trp1 MAL2-8cSUC2) was purchased from Scientific Research and Development GmbH (Oberursel, Germany). Stock cultures for *E. coli* were grown at 37°C and 200 rpm in Luria-Bertani (LB) medium and ampicillin (100 µg/mL). Stock cultures for *S. cerevisiae* were grown at 37°C and 200 rpm in 50 mL Corning tubes containing 10 mL of yeast extract-peptone-dextrose (YPD) medium with 20 g/L of dextrose, 20 g/L of peptone enzymatic digest of casein, and 10 g/L yeast extract. Main cultures were stored at −80°C in 1-mL microtubes with 20% of glycerol.

### Genes and plasmid construction

The metabolic pathway for the heterologous production of naringenin was defined based on the obtained results of the OptStoic-minRxn/minRxn algorithm, a problem of MILP aimed to identify the overall stoichiometry of conversion of L-tyrosine and L-phenylalanine to naringenin, by maximization of a desired yield using a metabolite/reaction database and their respective stoichiometric coefficients. For this, scientific information sources and databases such as Patric (45), MetaCyc (46), MetRxn (46), and Kyoto Encyclopedia of Genes and Genomes (KEGG) (47, 48) were used. The whole biosynthetic pathway is displayed in Fig. 1. Gene and enzyme selection criteria were previous evidence of expression in yeast (*S. cerevisiae* preferably), a low value of the Michaelis-Menten constant (*K_M_*) (obtained in BRENDA database), and the existence of mutations that can contribute to naringenin production. Hence, the set of genes was integrated by PAL/TAL from *R. capsulatus* (GenBank accession number: JX268036.1), 4 Cl from *S. lycopersicum* (GenBank accession number: NM_001346841)*,* CHS from *H. androsaemum* (GenBank accession number: AF315345), and CHI from *G. max* (GenBank accession number: AY595413). All DNA sequences were obtained from the National Center for Biotechnology Information (NCBI) GenBank.

**Fig 5:**
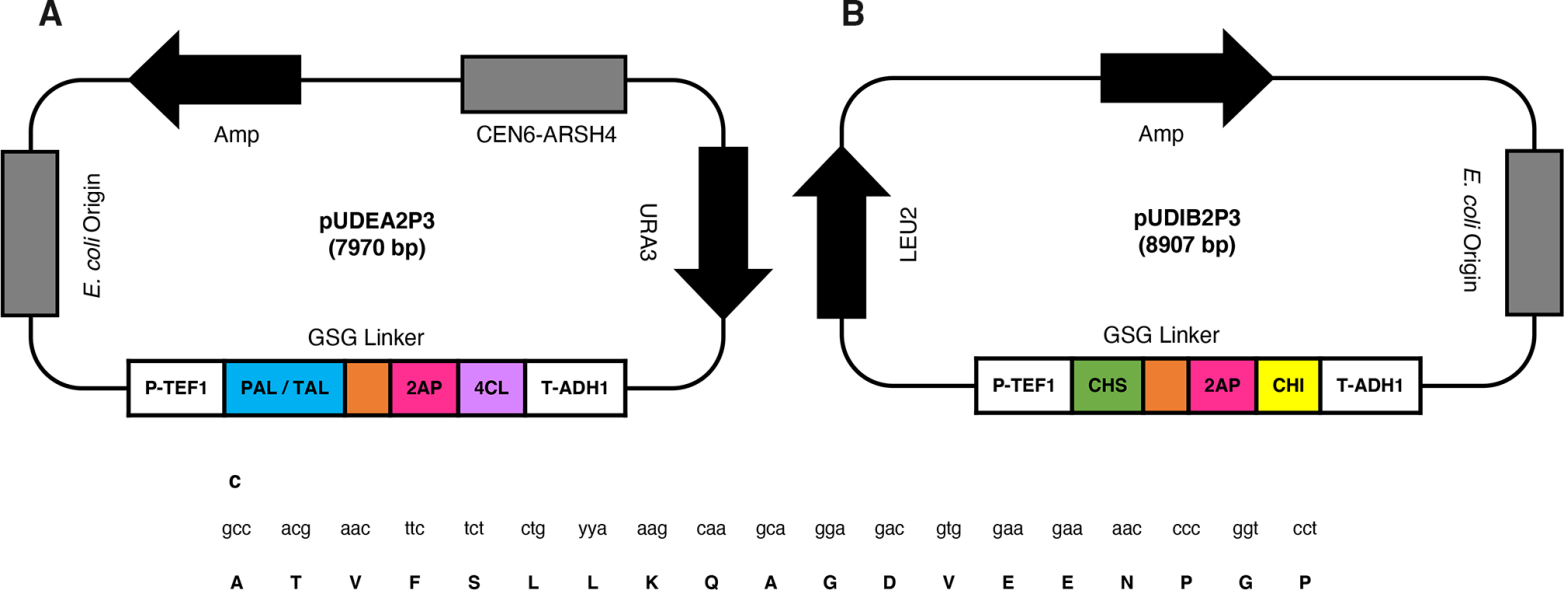
Constructs used in the expression of the phenylpropanoid pathway for naringenin production in *S. cerevisiae*. (**A**) pUDEA2P3 plasmid containing the PAL/TAL and the 4 Cl genes. (**B**) pUDIB2P3 plasmid containing the CHS and the CHI genes. Genes are indicated in color, the 2A peptide sequence is presented in red, GSG linker in brown, promotor/terminator in white, selection markers in black, and *E. coli* replication origin/recombination site are displayed in gray. (**C**) DNA and amino acid sequences of the PTV-1 2A peptide used.

The pUDEA2P3 and the pUDIB2P3 plasmids (Fig. 5) were selected and re-designated for this endeavor. In the first one (Fig. 5A), the genes of the PAL/TAL and 4 Cl were incorporated taking as a backbone the centromeric plasmid pUDE172 (GenBank accession number: JX268037.1), having URA3 as an auxotrophic marker (13). The pUDIB2P3 plasmid (Fig. 5B) was designated to contain the CHS and CHI genes, considering the plasmid pUDI065 (GenBank accession number: JX268039) with LEU2 as an auxotrophic marker (13). Each pair of genes in the plasmids were placed in a single cassette under a constitutive TEF1 promoter and ADH1 terminator; the genes were linked with the PTV-1 2A peptide sequence from porcine teschovirus-1 (15) (Fig. 2C). Both plasmids were synthesized by Gen Script (New Jersey, USA). Table 1 shows all the strains and vectors used in this study.

**TABLE 1:** Strains and vectors used in genetic engineering of *S. cerevisiae* for naringenin production

Strain or plasmid	Description	Reference
Strains		
*E. coli* BL21		This study
*S. cerevisiae*		
CEN.PK2-1C	Genotype MATalpha *ura3 his3 leu2 trp1* MAL2-8cSUC2	(13)
IM90	*S. cerevisiae* CEN.PK2-1C containing pUDEA2P3	This study
IM100	IM90 containing pUDIB2P3	This study
Plasmids		
pUDEA2P3	URA3, PAL/TAL, and 4 Cl genes with PTV-1 2A peptide sequence	13; this study
pUDIB2P3	LEU2, CHS, and CHI genes with PTV-1 2A peptide sequence	13; this study

### Plasmid construction and recombinant strain generation

The synthesized vectors were transformed and propagated in *E. coli* XL1-Blue. Each plasmid was isolated separately from *E. coli* using the Wizard Plus SV Minipreps DNA Purification System (Promega, Madison, WI), verifying the concentration and purity in a NanoDrop 1000 spectrophotometer (Thermo Fisher Scientific, Waltham, MA). The pUDEA2P3 plasmid was linearized using NcoI for its optimal integration in the chromosomal PYK2 locus. First, *S. cerevisiae* CEN.PK2-1C was transformed with pUDEA2P3 through the lithium acetate method (26). Briefly, *S. cerevisiae* competent cells were transformed with a mix containing PEG 3350, lithium acetate, ssDNA as a carrier, and the purified pUDEA2P3 plasmid at 42°C for 60 min. The cell suspension was incubated in 1 mL of sterile water at 30°C for 2 h and plated directly and in 1:10 and 1:100 dilutions per triplicate into the appropriate selection medium. The plates were incubated for 4 days at 30°C (26, 49), resulting in the IM90 strain. The transformation of the IM90 strain with the pUDIB2P3 plasmid was performed as previously described when constructing the *S. cerevisiae* IM100 strain. All transformations in *S. cerevisiae* with the plasmids described above were verified by colony PCR (50). After the genomic and plasmid DNA was extracted with the YeaStar Genomic DNA Kit (Zymo Research, Irvine, CA) and the Zymoprep Yeast Plasmid Miniprep I Kit (Zymo Research, Irvine, CA), respectively, gel electrophoresis was performed. The primers used for the verification of the PCR amplifications were carried out with 20–50 ng of DNA (corresponding to 10 µM of the forward and reverse primers) (Table 2) and 12.5 µL of Go Taq Master Mix (Promega, WI, USA) in a final volume of 25 µL. The reaction was performed in a Veriti 96-Well Thermal Cycler (Applied Biosystems, Singapore, Singapore), with the following parameters: (i) initial denaturation at 95°C for 2 min; (ii) 35 or 30 cycles of denaturation at 95°C for 30 s, primer annealing for 30 s at 65°C or 56°C depending on primer sets; and (iii) extension at 72°C for 1–2 min, with final elongation at 72°C for 5 min. Agarose gel separation of the amplicons was performed using a 1% (wt/vol) agarose (Sigma-Aldrich, Zwijdrecht, Netherlands) gel in 1 × Tris-acetate-EDTA (TAE) (40-mM Tris-acetate pH 8.0 and 1-mM EDTA).

**TABLE 2:** Primers used for verification

Name	Direction	Sequence	Purpose	Reference
FT90	Fw	CTCTAGGGTGTCGTTAATTACCC	Confirmation of first transformation	This study
FT91	Rev	CTGGCAAGGTAGACAAGCC		This study
ST100	Fw	GGCAGGTTTAGTTGAAAGAC	Confirmation of second transformation	This study
ST101	Rev	CGAAACAGTAAGAATGCAATGGC		This study

### Culture and media

The transformed and original strains of *S. cerevisiae* were cultured in Verduyn mineral medium containing 20 g/L of glucose, 5 g/L of ammonium sulfate, 3 g/L of potassium dihydrogen phosphate, 0.5 g/L of magnesium sulfate heptahydrate, trace element solution, and vitamin solution, adjusting the pH to 6.0 with potassium hydroxyde (KOH) or hydrochloric acid (HCl) and supplementing histidine monohydrochloride (1,034 mg/L), tryptophan (757.57 mg/L), leucine (3877. 55 mg/L), and uracil (757 mg/L) according to the auxotrophic requirements of the strains (13). The cultures were grown in 500-mL shake flasks with 100 mL of mineral medium at 30°C and 250 rpm in a MaxQ6000 incubator (Thermo Scientific, Marietta, USA). During fermentation, pH media was measured externally with an Orion Start potentiometer (Thermo Fisher Scientific, Waltham, MA).

### Biomass determination and substrate consumption estimation

Biomass was determined by diluting the cultures at 1:10 and by measuring optical density (OD) at 600 nm using a Genesys 10S UV-Vis spectrophotometer (Thermo Fisher Scientific, Waltham, MA). To start growth curves, OD was established at 0.2, inoculating from an intermediate culture in the exponential phase. The glucose consumption during the fermentation was monitored through soluble solid determination with a PR-101 Alfa Refractometer (Atago, Bellevue, USA).

### Naringenin analysis by HPLC

Culture medium supernatant (obtained by centrifugation at 9,000 *× g* for 8 min) was extracted with an equivalent volume of ethyl acetate (DEQ, Monterrey, Mexico) for 2 h at room temperature under dark conditions. After that, the organic upper phase was separated and evaporated in an EZ-2 series evaporator (Genevac, Gardiner, USA). The extract was resuspended using 1 mL of mobile phase A. Naringenin analysis was performed in HPLC with a diode array detector (Agilent 1290, Waldbronn, Germany) at 230 and 290 nm and a reversed-phase Zorbax SB-C18 column (4.6mm × 150 mm × 3.5 µm) kept at 30°C. Two mobile phases were used: Phase A was 20 mM of KH_2_PO_4_ (pH 2) with 1% acetonitrile, and Phase B was acetonitrile 100%. Gradient elution was performed at 1 mL/min, starting from 0 to 10% Phase B during 6 min, followed by a change from 10% to 40% of B during 17 min. From 23 to 27 min, B decreased to 0%; additionally, the gradient was held to 0% B for 10 min. A naringenin standard with 95% purity was purchased from Sigma Aldrich (St. Louis, MO, USA). The standard addition method was used for validating the identity of the analyte in the samples, adding naringenin at 2.5 µM.

## SUPPLEMENTAL MATERIAL

Fig. S1 and Fig. S2 spectrum.03374-23-s0001.docx Growth of transformed strains and electrophoresis validation.

ASM does not own the copyrights to Supplemental Material that may be linked to, or accessed through, an article. The authors have granted ASM a non-exclusive, world-wide license to publish the Supplemental Material files. Please contact the corresponding author directly for reuse.
