# Supplementary material for: Saccharomyces cerevisiae biofactory to produce naringenin using a systems biology approach and a bicistronic vector expression strategy in flavonoid production
Source: Microbiol Spectr. 2023 Dec 13;12(1):e03374-23. doi: 10.1128/spectrum.03374-23 (PMC10871697; doi:10.1128/spectrum.03374-23)
Supplement: Fig. S1 and Fig. S2 — Growth of transformed strains and electrophoresis validation. [file spectrum.03374-23-s0001.docx]

**SUPPLEMENTAL MATERIAL**

**FIG S1** Representative culture plates with selection media showing the growth of *S. cerevisiae* strains transformed and comprising the naringenin biosynthetic pathway. **a** The IM90 strain harbors the integrative cassette for the PAL/TAL and 4CL enzymes (top-left), negative control without the pUDEA2P3 plasmid (center-left), and the negative control without plasmid and ssDNA carrier (bottom-left). **b** The IM100 strain harbors the pUDIB2P3 plasmid that codifies for the CHS and CHI enzymes (top-right), the negative control without the pUDIB2P3 plasmid (center-right), and negative control without plasmid and ssDNA carrier (bottom-right).

**FIG S2** Gel electrophoresis validation for pUDEA2P3 and pUDIB2P3 sequential transformation of *S. cerevisiae* for naringenin production. **a** Gel electrophoresis (0.1% agarose) of the amplified fragment (2.2 kb) by PCR in the first transformation. **b** Gel electrophoresis of the amplified fragment (0.65 kb) obtained by PCR in the second transformation. C#P# notation refers to the colony and plate number taken from solid selection media for molecular analysis.
